# Antibacterial Alkaloids and Polyketide Derivatives from the Deep Sea-Derived Fungus *Penicillium cyclopium* SD-413

**DOI:** 10.3390/md18110553

**Published:** 2020-11-06

**Authors:** Yan-He Li, Xiao-Ming Li, Xin Li, Sui-Qun Yang, Xiao-Shan Shi, Hong-Lei Li, Bin-Gui Wang

**Affiliations:** 1 Key Laboratory of Experimental Marine Biology, Institute of Oceanology, Chinese Academy of Sciences, Nanhai Road 7, Qingdao 266071, China; liyanhe@qdio.ac.cn (Y.-H.L.); lixmqd@qdio.ac.cn (X.-M.L.); lixin@qdio.ac.cn (X.L.); yangsuiqun@qdio.ac.cn (S.-Q.Y.); Shixs@qdio.ac.cn (X.-S.S.); 2 Laboratory of Marine Biology and Biotechnology, Qingdao National Laboratory for Marine Science and Technology, Wenhai Road 1, Qingdao 266237, China; 3 College of Marine Sciences, University of Chinese Academy of Sciences, Yuquan Road 19A, Beijing 100049, China; 4 Center for Ocean Mega-Science, Chinese Academy of Sciences, Nanhai Road 7, Qingdao 266071, China

**Keywords:** *Penicillium cyclopium*, marine sediment-derived fungus, quinazoline alkaloid, polyketide derivatives, antibacterial activity

## Abstract

Nine secondary metabolites (**1**–**9**), including two new polyketide derivatives 9-dehydroxysargassopenilline A (**4**) and 1,2-didehydropeaurantiogriseol E (**5**), along with seven known related secondary metabolites (**1**–**3** and **6**–**9**), were isolated and identified from the deep sea-derived fungus *Penicillium* *cyclopium* SD-413. Their structures were elucidated on the basis of 1D/2D NMR spectroscopic and mass spectrometric analysis and the absolute configurations were determined by the combination of NOESY correlations and time-dependent density functional (TDDFT) ECD calculations. Compounds **1**–**9** inhibited some pathogenic bacteria including *Escherichia coli*, *E. ictaluri*, *Edwardsiella tarda*, *Micrococcus luteus*, *Vibrio anguillarum*, and *V. harveyi*, with MIC (minimum inhibitory concentration) values ranging from 4 to 32 μg/mL.

## 1. Introduction

Marine sediment-derived fungi have received considerable attention as a valuable resource of bioactive metabolites with diversified chemical structures including alkaloids, peptides, polyketides, and terpenoids [1,2,3,4,5,6]. These natural products are characterized by intriguing biological properties such as anticancer, antifouling, antimicrobial, antioxidant, and antiviral activities [4,5,6,7,8,9].

**Figure 1:**
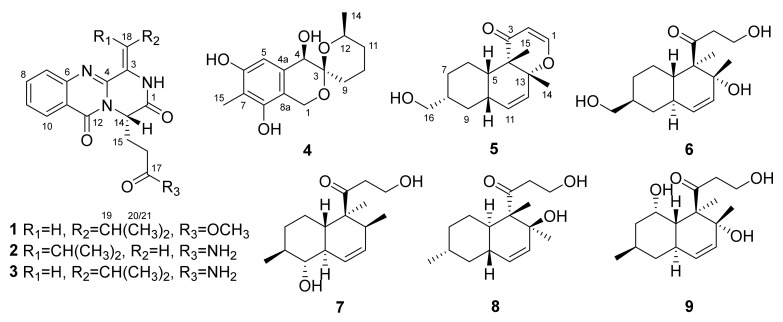
Structures of compounds **1**–**9**.

In our continuing excavation to identify new bioactive metabolites from deep sea-derived fungi [7,8,9,10,11,12], the fungus *Penicillium cyclopium* SD-413, which was obtained from a sediment sample collected from the East China Sea, was screened out for chemical investigations. As a result, two new polyketide derivatives, 9-dehydroxysargassopenilline A (**4**) and 1,2-didehydropeaurantiogriseol E (**5**), along with seven known related metabolites (**1**–**3** and **6**–**9**) (Figure 1), were isolated and identified from the culture extract of the fungus. Chemical structures of the isolated compounds were established by detailed interpretation of 1D/2D NMR spectroscopic and mass spectrometric data and the absolute configurations of compounds **4** and **5** were determined by ECD calculations. All of these compounds were evaluated for antibacterial activities against some human and fish pathogenic bacteria. Herein, the details of isolation, structure elucidation, and biological activities of compounds **1**–**9** are described.

## 2. Results and Discussion

### 2.1. Structure Elucidation of the Isolated Compounds

Compounds **1** and **2** were originally treated as new quinazoline alkaloids during the preparation of this manuscript, and their structures were elucidated by detailed analysis of NMR spectroscopic and high-resolution mass spectrometric data. However, when the manuscript was ready for submission, Cao and co-workers reported three new quinazoline-containing diketopiperazines, namely polonimides A–C, from the marine-derived fungus *Penicillium polonicum* HBU-114 [13]. Among them, polonimides A and B had virtually identical NMR data to those of compounds **1** and **2**, respectively. The NMR data of polonimides A and B were recorded on a Bruker AV-600 spectrometer, whereas for compounds **1** and **2**, the data were acquired on a Bruker Avance 500 spectrometer. For future reference, our NMR data of compounds **1** and **2** are listed in the Supporting Information (Figures S1–S11, Table S1).

**Figure 2:**
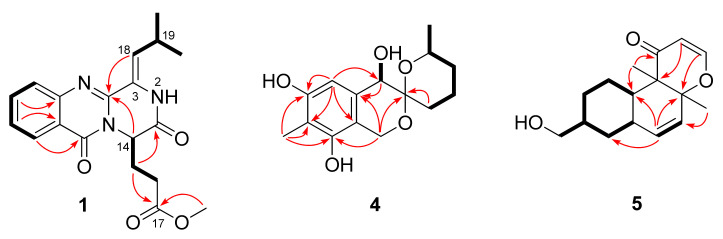
Key COSY (bold lines) and HMBC (arrows) correlations of compounds **1**, **4**, and **5**.

The molecular formula of 9-dehydroxysargassopenilline A (**4**) was determined to be C_15_H_20_O_5_ on the basis of HRESIMS data (six degrees of unsaturation, Figure S12). The NMR spectroscopic data (Table 1, Figures S13 and S14) revealed the presence of 15 carbon atoms, which were clarified into two methyls, four methylenes, three methines, and six non-protonated carbons. Extensive analysis of its ^1^H and ^13^C NMR data revealed that compound **4** is a derivative of 6,6-spiroketal, very similar to sargassopenilline A, which was previously isolated from the axenic culture of the marine alga-derived fungus *Penicillium thomii* KMM4645 [14]. However, signals at *δ*_H_ 4.15 and *δ*_C_ 66.1 for an oxygenated methine (CH-9) in the NMR spectra of sargassopenilline A disappeared in those of compound **4**, while resonances for an additional methylene at *δ*_H_ 1.40/1.94 and *δ*_C_ 29.4 were observed in the NMR spectra of compound **4** (Table 1). The above observation disclosed that the oxymethine (CH-9) of sargassopenilline A was replaced by a methylene group in compound **4**. This deduction was further verified by the proton-proton spin-coupling system from C-9 to C-12 and C-14 established by the COSY and HSQC analysis of compound **4** (Figures S15 and S16), as well as by the key HMBC correlations from H-1 and H_2_-9 to C-3 (Figure 2 and  Figure S17). Thus, the planar structure of compound **4** was determined as shown in Figure 1.

**Figure 3:**
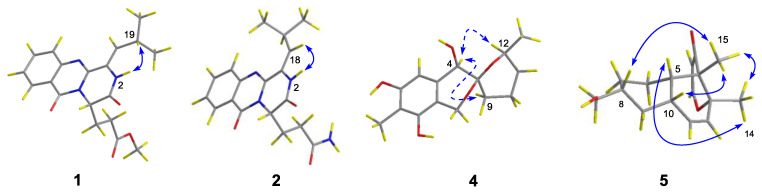
Key NOESY correlations for **1**, **2**, **4**, and **5** (solid lines: *β*-orientation; dashed lines: *α*-orientation).

**Figure 4:**
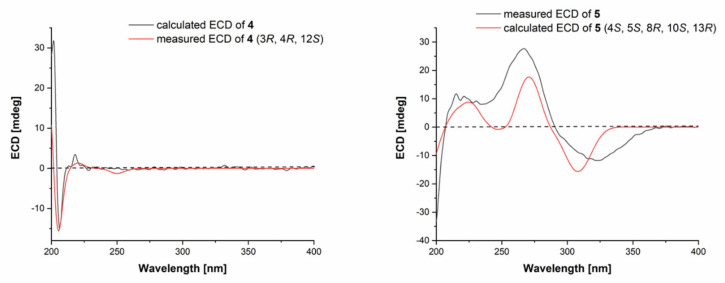
Experimental and calculated ECD spectra of compounds **4** and **5**.

The relative configuration of compound **4** was assigned by analysis of NOESY data (Figure 3 and Figure S18). The key NOE correlations from H-4 to H-9*α* and H-12 oriented these protons to the same face of the molecule. The absolute configuration of compound **4** was established by the TDDFT-ECD calculation in Gaussian 09 [15]. After geometry optimization, the minimum energy conformers were obtained, and then the TDDFT method at the B3LYP/6-31G level was employed to generate the calculated ECD spectrum of compound **4** (Figure 4). The experimental ECD spectrum of compound **4** exhibited excellent accordance with that calculated for the absolute configuration (3*R*, 4*R*, 12*S*) in compound **4** (Figure 4), which established the absolute configuration of compound **4**.

1,2-Didehydropeaurantiogriseol E (**5**) was isolated as a colorless oil and its HRESIMS data (Figure S19) gave the molecular formula as C_16_H_22_O_3_. The ^1^H and ^13^C NMR data (Table 1, Figures S20 and S21) of compound **5** showed a close relationship to that of peaurantiogriseol E, a derivative of polyketide isolated from *Penicillium aurantiogriseum* [16]. However, signals for two methylenes (C-1 and C-2) in the NMR spectra of peaurantiogriseol E were not present in those of compound **5**. Instead, two olefinic methines at *δ*_C/H_ 157.9/7.26 (CH-1) and *δ*_C/H_ 105.6/5.19 (CH-2) were observed in the NMR spectra of compound **5** (Figures S20–S24). Furthermore, the HMBC correlations (Figure 2 and Figure S24) from H-1 to C-13 and from H-2 to C-4 revealed the presence of dihydropyranone ring in compound **5**.

The relative configuration of compound **5** was established by analysis of NOESY data (Figure 3 and Figure S25). The key NOE correlations from H-14 to H-5 and H-15 and from H-15 to H-8 and H-10 located these groups on the same side of the molecule. Similarly, the absolute configuration of compound **5** was determined by the TDDFT-ECD calculation in Gaussian 09 [15]. The TDDFT-ECD spectrum calculated at the B3LYP/6-31G level for the isomer (4*S*, 5*S*, 8*R*, 10*S*, 13*R*) of compound **5** matched well with the experimental ECD spectrum (Figure 4).

In addition to the two new secondary metabolites (**4** and **5**) and three known quinazoline alkaloids (**1**–**3**), four known derivatives of polyketide (**6**–**9**) were also isolated from the culture extract of the fungus *P. cyclopium* SD-413. By detailed spectroscopic analysis as well as comparisons with reported data, the structures of compounds **3** and **6**–**9** were identified as aurantiomide C (**3**) [17], craterellone D (**6**) [18], peaurantiogriseol A (**7**) [16], aspermytin A (**8**) [19], and 1-(2,8-dihydroxy-1,2,6-trimethyl-1,2,6,7,8,8a-hexahydronaphthalen-1-yl)-3-hydroxy-1-propanone (**9**) [20], respectively.

### 2.2. Antibacterial Activities of the Isolated Compounds

The obtained compounds **1**–**9** were evaluated for antibacterial activities against 2 human pathogenic bacteria (*Escherichia coli* and *Staphylococcus aureus*) and 10 fish pathogenic bacteria (*Aeromonas hydrophila*, *Edwardsiella ictaluri*, *E. tarda*, *Micrococcus luteus*, *Pseudomonas aeruginosa*, *Vibrio alginolyticus*, *V. anguillarum, V. harveyi*, *V. parahemolyticus*, and *V. vulnificus*). Compounds **1**–**3** exhibited inhibitory activities against *E. coli*, *E. ictaluri*, *E. tarda*, and *V. harveyi*, with MIC values ranging from 4.0 to 32 μg/mL (Table 2), while compound **4** showed potent inhibitory activity against *M. luteus,* with an MIC value of 4.0 μg/mL, and compound **5** showed potent activities against *V. anguillarum* and *V. harveyi*, each with an MIC value of 4.0 μg/mL. These results indicated that the formation of dihydropyranone ring (**5** vs. **6**–**9**) in structures strengthened their effects against *V. anguillarum* and *V. harveyi*. However, the tested compounds were inactive against the remaining microorganisms.

## 3. Experimental Section

### 3.1. General Experimental Procedures

Optical rotations: an Optical Activity AA-55 polarimeter (Optical Activity Ltd., Cambridgeshire, UK); UV spectra: a PuXi TU-1810 UV-visible spectrophotometer (Shanghai Lengguang Technology Co. Ltd., Shanghai, China); ECD spectra: a JASCO J-715 spectropolarimeter (JASCO, Tokyo, Japan); NMR spectra: a Bruker Avance 500 spectrometer (Bruker Biospin Group, Karlsruhe, Germany); mass spectra: an API QSTAR Pulsar 1 mass spectrometer (Applied Biosystems, Foster City, CA, USA); analytical HPLC: a Dionex HPLC system, equipped with P680 pump (Dionex, Sunnyvale, CA, USA); and TLC: silica gel GF254 precoated plates (Qingdao Haiyang Chemical Group Corporation, Qingdao, China). Column chromatography (CC): 100–200 mesh and 200–300 mesh silica gel (SiO_2_; Qingdao Haiyang Chemical Group Corporation), 40–63 μm RP-18 reverse-phase Si gel (Merck, Darmstadt, Germany), and Sephadex LH-20 (Merck, Darmstadt, Germany). All solvents were distilled prior to use.

### 3.2. Fungal Material

The fungus *Penicillium cyclopium* SD-413 was isolated from a marine sediment sample collected from the East China Sea in May 2017. It was identified using a molecular biological protocol described in our previous report [21] by DNA amplification and the sequencing of the ITS (internal transcribed spacer) region. The sequenced data derived from the fungal strain were deposited in GenBank (accession no. MN818582). A BLAST search result showed that the sequence was most similar (99%) to the sequence of *Penicillium cyclopium* (accession no. MT990551.1). The strain is preserved at the Key Laboratory of Experimental Marine Biology, Institute of Oceanology, Chinese Academy of Sciences (IOCAS).

### 3.3. Fermentation

For the purpose of chemical composition analysis, the fresh mycelia of *P. cyclopium* SD-413 were grown on PDA (potato dextrose agar) medium at 28 °C for five days and then cultivated in a 1 L conical flask (100 flasks) with solid rice medium (each flask contained 70 g rice; 0.1 g corn flour; 0.3 g peptone; 0.1 g sodium glutamate; and 100 mL naturally sourced and filtered seawater, which was obtained from the Huiquan Gulf of the Yellow Sea near the campus of Institute of Oceanology, Chinese Academy of Sciences (IOCAS), pH 6.5–7.0) for 30 days at room temperature.

### 3.4. Extraction and Isolation

The whole fermented cultures were extracted four times with EtOAc. The solvents were evaporated under reduced pressure to yield an organic extract (71.2 g), which was fractionated by vacuum liquid chromatography (VLC) on silica gel eluting with different solvents of increasing polarity from petroleum ether (PE) to MeOH to yield nine fractions (Frs. 1–9) based on TLC and HPLC analysis. Fr. 5 (6.65 g), eluted with PE-EtOAc (2:1), was further purified by CC over Lobar LiChroprep RP-18 with a MeOH-H_2_O gradient (from 10:90 to 100:0) to yield ten subfractions (Fr. 5.1–5.10). Fr. 5.6 (126.1 mg) was further purified by CC on Sephadex LH-20 (MeOH) and then by semi-preparative HPLC (55% MeOH-H_2_O, 5 mL/min) to yield compounds **3** (5.9 mg, *t*_R_ 22.5 min) and **4** (20.7 mg, *t*_R_ 27.3 min). Fr. 7 (23.52 g), eluted with CH_2_Cl_2_-MeOH (20:1), was further purified by CC on silica gel, eluting with a PE-EtOAc gradient (from 5:1 to 2:1), to yield two subfractions (Fr. 7.1 and Fr. 7.2). Fr. 7.1 (12.91 g) was further purified by CC over Lobar LiChroprep RP-18 with a MeOH-H_2_O gradient (from 10:90 to 100:0) to yield ten subfractions (Fr. 7.1.1–7.1.10). Fr. 7.1.6 (100.9 mg) was purified by CC on silica gel eluting with a CH_2_Cl_2_-MeOH gradient (from 200:1 to 20:1), and then by CC on Sephadex LH-20 (MeOH), obtained compounds **6** (16.1 mg) and **7** (10.7 mg). Fr. 7.1.7 was further purified by CC over RP-18 eluting with a MeOH-H_2_O gradient (10:90 to 100:0) and by semi-preparative HPLC (MeOH-H_2_O, 60% to 85%, 5 mL/min) to obtain **1** (8.3 mg, *t_R_* 18.1 min), **2** (8.2 mg, *t_R_* 22.3 min), and **5** (29.3 mg, *t_R_* 26.7 min). Fr. 7.2 (10.42 g) was further purified by CC over Lobar LiChroprep RP-18 with a MeOH-H_2_O gradient (from 10:90 to 100:0) to yield ten subfractions (Fr. 7.2.1–7.2.10). Fr. 7.2.3 (201.3 mg) was purified by CC on silica gel eluting with a CH_2_Cl_2_-MeOH gradient (from 200:1 to 20:1) and then by CC on Sephadex LH-20 (MeOH) to obtain compounds **8** (64.6 mg) and **9** (12.9 mg).

Polonimide A (**1**): Amorphous powder; [α${]}_{D}^{20}$ = +67.3 (*c* 0.10, CHCl_3_); UV (MeOH) *λ*_max_ (log *ε*) 212 (7.32), 312 (4.48) nm; ECD (0.33 mg/mL, MeOH) *λ*_max_ (Δ*ε*) 227 (+25.18), 250 (−16.35), 274 (−3.28), 295 (−6.27), 329 (+4.20) nm; ^1^H and ^13^C NMR data, Table S1; HRESIMS *m/z* 356.1609 [M + H]^+^ (calcd for C_19_H_22_N_3_O_4_, 356.1607), 378.1438 [M + Na]^+^ (calcd for C_19_H_21_N_3_O_4_Na, 378.1436).

Polonimide B (**2**): Amorphous powder; [α${]}_{D}^{20}$ = +14.2 (*c* 0.15, CHCl_3_); UV (MeOH) *λ*_max_ (log *ε*) 212 (7.32), 312 (4.48) nm; ECD (0.26 mg/mL, MeOH) *λ*_max_ (Δ*ε*) 223 (+21.59), 246 (−18.10), 271 (−1.34), 295 (−5.81), 326 (+6.74) nm; ^1^H and ^13^C NMR data, Table S1; HRESIMS *m/z* 341.1609 [M + H]^+^ (calcd for C_18_H_21_N_4_O_3_, 341.1608).

9-Dehydroxysargassopenilline A (**4**): Amorphous powder; [α${]}_{D}^{20}$ = −83.5 (*c* 0.10, MeOH); UV (MeOH) *λ*_max_ (log *ε*) 235 (3.25), 282 (3.10) nm; ECD (7.21 mM, MeOH) *λ*_max_ (Δ*ε*) 202 (+31.83), 206 (−17.93), 218 (+3.45), 229 (−0.51) nm; ^1^H and ^13^C NMR data, Table 1; HRESIMS *m/z* 279.1236 [M + H]^+^ (calcd for C_15_H_19_O_5_, 279.1238).

1,2-Didehydropeaurantiogriseol E (**5**): Amorphous powder; [α${]}_{D}^{20}$ = −40.3 (*c* 0.12, MeOH); UV (MeOH) *λ*_max_ (log *ε*) 201 (2.49), 254 (1.95) nm; ECD (7.11 mM, MeOH) *λ*_max_ (Δ*ε*) 215 (+11.73), 235 (+8.04), 267 (+27.67), 324 (−11.74) nm; ^1^H and ^13^C NMR data, Table 1; HRESIMS *m/z* 263.1644 [M + H]^+^ (calcd for C_16_H_23_O_3_, 263.1642).

### 3.5. Antibacterial Assays

Antibacterial evaluation against human pathogenic bacteria (*Escherichia coli* and *Staphylococcus aureus*) and fish pathogenic bacteria (*Aeromonas hydrophila*, *Edwardsiella ictaluri*, *E. tarda*, *Micrococcus luteus*, *Pseudomonas aeruginosa*, *Vibrio alginolyticus*, *V. anguillarum*, *V. harveyi*, *V. parahemolyticus*, and *V. vulnificus*) was carried out by the microplate assay with a microplate assay with three repetitions [22]. The bacteria including *A. hydrophila*, *E. ictaluri*, *E. coli*, *E. tarda*, *M. luteus*, *S. aureus*, *V. harveyi*, and *V. parahemolyticus* were incubated in LB medium (1% peptone, 1% NaCl, 0.5% yeast extract powder in distilled water), while the rest of the bacteria in assay were incubated in TSB medium (1.5% tryptone, 0.5% soytone, 0.5% NaCl in distilled water). The pathogenic bacteria and fish pathogenic strains were provided by the Institute of Oceanology, Chinese Academy of Sciences. Chloramphenicol was used as positive control against bacteria.

### 3.6. Computational Section

Conformational searches were performed via molecular mechanics using the MM+ method in HyperChem software (Version 8.0, Hypercube, Inc., Gainesville, FL, USA), and the geometries were further optimized at B3LYP/6-31G(d) level via Gaussian 09 software (Version D.01; Gaussian, Inc.: Wallingford, CT, USA) [15] to give the energy-minimized conformers. After that, the optimized conformers were subjected to the calculations of ECD spectra using TDDFT at B3LYP/6-31G level. Solvent effects of the MeCN solution were evaluated at the same DFT (density functional theory) level using the SCRF/PCM (polarizable continuum model) method.

## 4. Conclusions

In summary, chemical investigations of the deep sea-derived fungus *P. cyclopium* SD-413 provided two new polyketide derivatives, 9-dehydroxysargassopenilline A (**4**) and 1,2-didehydropeaurantiogriseol E (**5**), along with seven known related secondary metabolites (**1**–**3** and **6**–**9**). The structures of these compounds were elucidated on the basis of NMR spectroscopic and mass spectrometric analysis and the absolute configurations of compounds **4** and **5** were established by the TDDFT-ECD calculation. Compounds **1**–**5** exhibited inhibitory activities against some tested human and fish pathogenic bacteria, with MIC values ranging from 4.0 to 32 μg/mL. These compounds might be used as leading compounds for the development of agents against the pathogenic bacteria.

## Figures and Tables

**Table 1:** ^1^H and ^13^C NMR data of compounds **4** and **5** (measured in DMSO-*d*_6_).

No.	4		No.	5	
	*δ*_H_ (*J* in Hz) ^a^	*δ*_C_, Type ^b^		*δ*_H_ (*J* in Hz) ^a^	*δ*_C_, Type ^b^
1*α*	4.34, d (14.7)	58.2, CH_2_	1	7.26, d (5.9)	157.9, CH
1*β*	4.49, d (14.7)		2	5.19, d (5.9)	105.6, CH
3		95.7, C	3		199.0, C
4	4.09, d (8.5)	69.6, CH	4		47.4, C
4a		133.8, C	5	1.59, m	42.2, CH
5	6.55, s	104.4, CH	6*α*	1.13, m	26.4, CH_2_
6		154.3, C	6*β*	2.75, m	
7		108.9, C	7*α*	1.00, m	29.4, CH_2_
8		149.7, C	7*β*	1.77, m	
8a		112.6, C	8	1.48, m	40.7, CH
9*α*	1.40, m	29.4, CH_2_	9*α*	0.81, m	35.0, CH_2_
9*β*	1.94, m		9*β*	1.85, m	
10*α*	1.61, m	18.5, CH_2_	10	1.89, m	40.1, CH
10*β*	1.80, m		11	5.48, dd (10.1, 2.1)	131.4, CH
11*α*	1.11, m	31.9, CH_2_	12	5.67, dd (10.1, 2.1)	128.3, CH
11*β*	1.55, m		13		85.3, C
12	3.75, m	66.9, CH	14	1.37, s	21.6, CH_3_
14	1.01, d (6.3)	21.6, CH_3_	15	1.15, s	13.9, CH_3_
15	1.95, s	8.6, CH_3_	16	3.22, m	66.3, CH_2_
4-OH	4.57, d (8.5)		16-OH	4.42, br	
6-OH	8.93, s				
8-OH	8.08, s				

^a^ Measured at 500 MHz; ^b^ measured at 125 MHz.

**Table 2:** Antibacterial activities of compounds **1**–**9** (MIC, μg/mL).

Strains	1	2	3	4	5	6	7	8	9	Chl ^a^
*Escherichia coli*	8.0	4.0	8.0	n.a.	16	8.0	n.a.	32	16	2.0
*Edwardsiella tarda*	8.0	8.0	8.0	16	n.a.	32	16	32	8.0	2.0
*Edwardsiella ictaluri*	8.0	8.0	8.0	n.a.	n.a.	n.a.	32	16	n.a.	0.5
*Micrococcus luteus*	32	n.a.	n.a.	4.0	32	n.a.	32	n.a.	n.a.	2.0
*Vibrio harveyi*	8.0	8.0	32	n.a.	4.0	8.0	n.a.	32	32	0.5
*Vibrio anguillarum*	n.a.	n.a.	n.a.	32	4.0	32	16	32	n.a.	1.0

^a^ Chl: chloramphenicol (positive control); n.a.: no activity (MIC > 64 μg/mL).

## Supplementary Materials

The following are available online at https://www.mdpi.com/1660-3397/18/11/553/s1. Figure S1: HRESIMS spectrum of compound **1**. Figure S2: ^1^H NMR (500 MHz, DMSO-*d*_6_) spectrum of compound **1**. Figure S3: ^13^C NMR (125 MHz, DMSO-*d*_6_) and DEPT spectra of compound **1**. Figure S4: COSY spectrum of compound **1**. Figure S5: HSQC spectrum of compound **1**. Figure S6: HMBC spectrum of compound **1**. Figure S7: NOESY spectrum of compound **1**. Figure S8: HRESIMS spectrum of compound **2**. Figure S9: ^1^H NMR (500 MHz, DMSO-*d*_6_) spectrum of compound **2**. Figure S10: ^13^C NMR (125 MHz, DMSO-*d*_6_) and DEPT spectra of compound **2**. Figure S11: NOESY spectrum of compound **2**. Table S1: ^1^H and ^13^C NMR data of compounds **1** and **2**. Figure S12: HRESIMS spectrum of compound **4**. Figure S13: ^1^H NMR (500 MHz, DMSO-*d*_6_) spectrum of compound **4**. Figure S14: ^13^C NMR (125 MHz, DMSO-*d*_6_) and DEPT spectra of compound **4**. Figure S15: COSY spectrum of compound **4**. Figure S16: HSQC spectrum of compound **4**. Figure S17: HMBC spectrum of compound **4**. Figure S18: NOESY spectrum of compound **4**. Figure S19: HRESIMS spectrum of compound **5**. Figure S20. ^1^H NMR (500 MHz, DMSO-*d*_6_) spectrum of compound **5**. Figure S21: ^13^C NMR (125 MHz, DMSO-*d*_6_) and DEPT spectra of compound **5**. Figure S22: COSY spectrum of compound **5**. Figure S23: HSQC spectrum of compound **5**. Figure S24: HMBC spectrum of compound **5**. Figure S25: NOESY spectrum of compound **5**.
