# Supplementary material for: Antibacterial Alkaloids and Polyketide Derivatives from the Deep Sea-Derived Fungus Penicillium cyclopium SD-413
Source: Mar Drugs. 2020 Nov 6;18(11):553. doi: 10.3390/md18110553 (PMC7695020; doi:10.3390/md18110553)

Supplementary File

for

Antibacterial Alkaloids and Polyketide Derivatives from the Deep Sea-Derived Fungus *Penicillium cyclopium* SD-413

Yan-He Li [1,2,3], Xiao-Ming Li [1,2,4], Xin Li [1,2,4], Sui-Qun Yang [1,2,4], Xiao-Shan Shi [1,2,4], and Hong-Lei Li [1,2,4,*] and **Bin-Gui Wang [1,2,3,4,*]**

[1] Key Laboratory of Experimental Marine Biology, Institute of Oceanology, Chinese Academy of Sciences, Nanhai Road 7, Qingdao 266071, China; E-Mails: liyanhe@qdio.ac.cn (Y.-H.L.); lixmqd@qdio.ac.cn (X.-M.L.); yangsuiqun@qdio.ac.cn (S.-Q.Y.); Shixs@qdio.ac.cn (X.-S.S.); lixin@qdio.ac.cn (X.L.)

[2] Laboratory of Marine Biology and Biotechnology, Qingdao National Laboratory for Marine Science and Technology, Wenhai Road 1, Qingdao 266237, China

[3] College of Marine Sciences, University of Chinese Academy of Sciences, Yuquan Road 19A, Beijing 100049, China

[4] Center for Ocean Mega-Science, Chinese Academy of Sciences, Nanhai Road 7, Qingdao 266071, China

* Correspondence lihonglei@qdio.ac.cn (H.-L.L.); wangbg@ms.qdio.ac.cn (B.-G.W.); Tel.: +86-532-8289-8553 (B.-G.W.)

Content

Figure S1. HRESIMS spectrum of compound **1**.

Figure S2. ^{1}H NMR (500 MHz, DMSO-d_6) spectrum of compound **1**.

Figure S3. ^{13}C NMR (125 MHz, DMSO-d_6) and DEPT spectra of compound **1**.

Figure S4. COSY spectrum of compound **1**.

Figure S5. HSQC spectrum of compound **1**.

Figure S6. HMBC spectrum of compound **1**.

Figure S7. NOESY spectrum of compound **1**.

Figure S8. HRESIMS spectrum of compound **2**.

Figure S9. ^{1}H NMR (500 MHz, DMSO-d_6) spectrum of compound **2**.

Figure S10. ^{13}C NMR (125 MHz, DMSO-d_6) and DEPT spectra of compound **2**.

Figure S11. NOESY spectrum of compound **2**.

Table S1. ^{1}H and ^{13}C NMR data of compounds **1** and **2**.

Figure S12. HRESIMS spectrum of compound **4**.

Figure S13. ^{1}H NMR (500 MHz, DMSO-d_6) spectrum of compound **4**.

Figure S14. ^{13}C NMR (125 MHz, DMSO-d_6) and DEPT spectra of compound **4**.

Figure S15. COSY spectrum of compound **4**.

Figure S16. HSQC spectrum of compound **4**.

Figure S17. HMBC spectrum of compound **4**.

Figure S18. NOESY spectrum of compound **4**.

Figure S19. HRESIMS spectrum of compound **5**.

Figure S20. ^{1}H NMR (500 MHz, DMSO-d_6) spectrum of compound **5**.

Figure S21. ^{13}C NMR (125 MHz, DMSO-d_6) and DEPT spectra of compound **5**.

Figure S22. COSY spectrum of compound **5**.

Figure S23. HSQC spectrum of compound **5**.

Figure S24. HMBC spectrum of compound **5**.

Figure S25. NOESY spectrum of compound **5**.

Figure S1. HRESIMS spectrum of compound **1**.

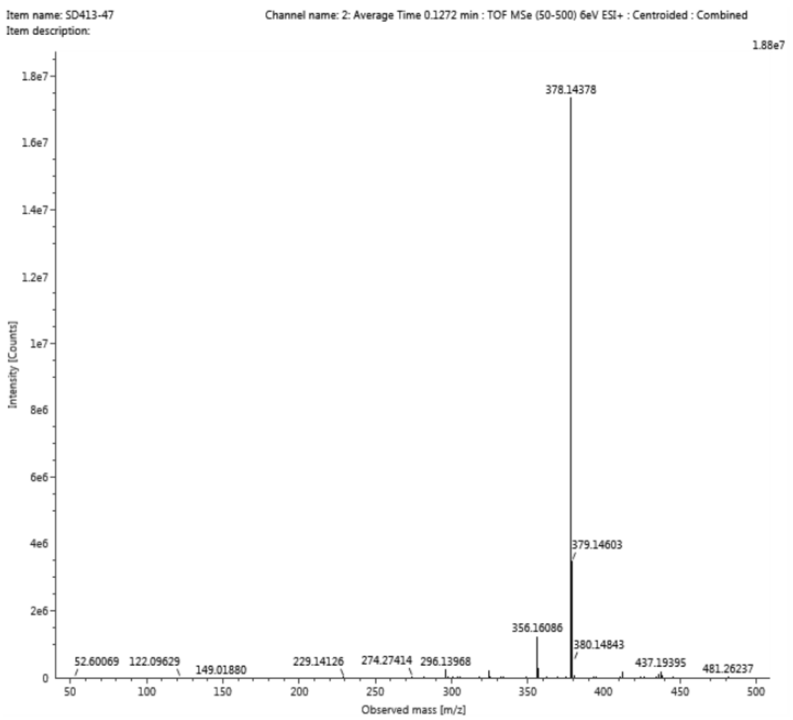

Figure S2. ^{1}H NMR (500 MHz, DMSO-d_6) spectrum of compound **1**.

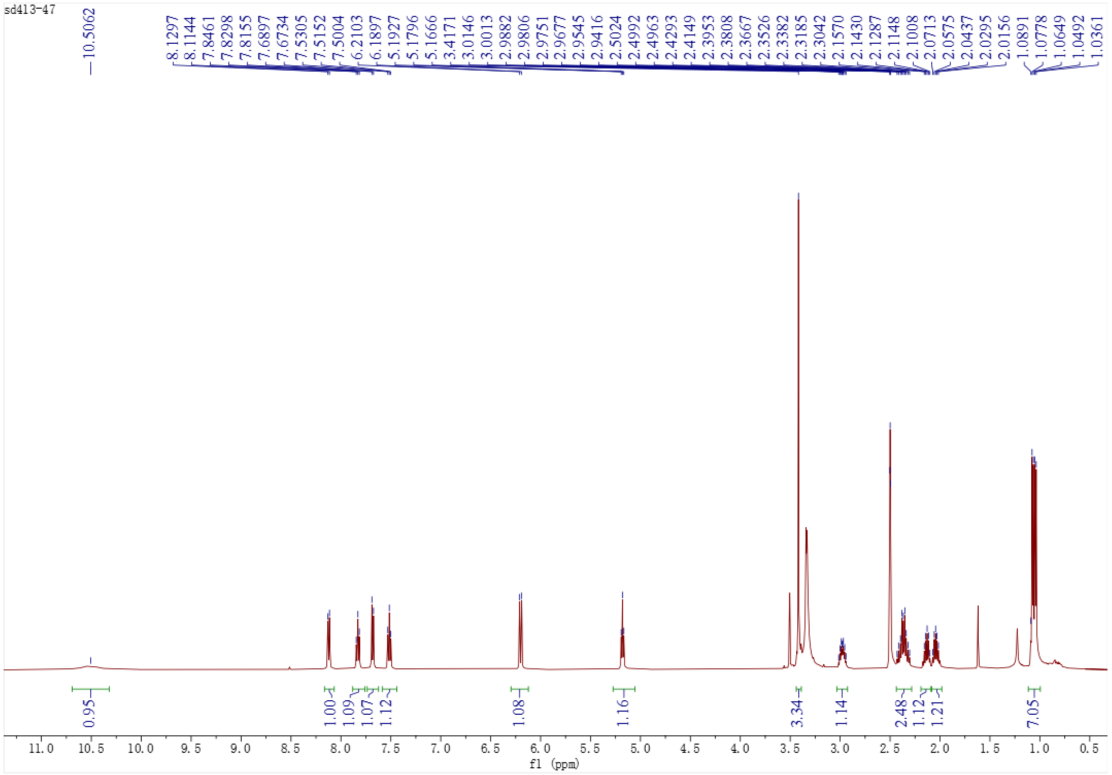

Figure S3. ^{13}C NMR (125 MHz, DMSO-d_6) and DEPT spectra of compound **1**.

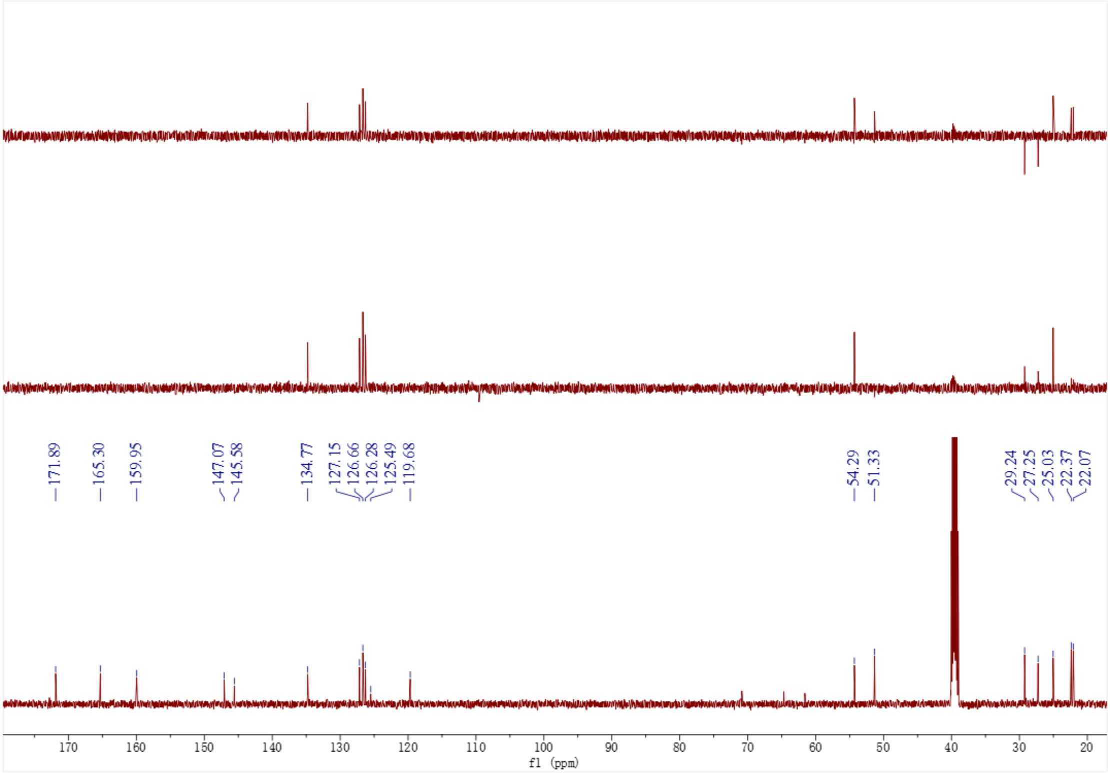

Figure S4. COSY spectrum of compound **1**.

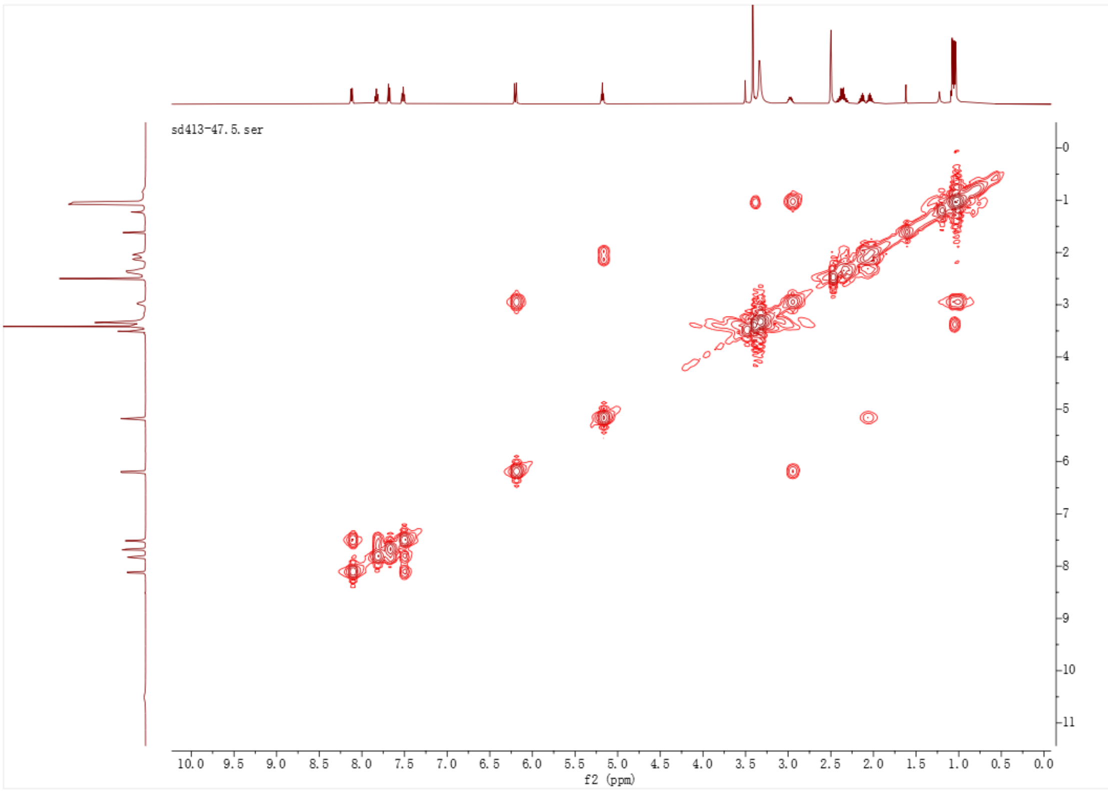

Figure S5. HSQC spectrum of compound **1**.

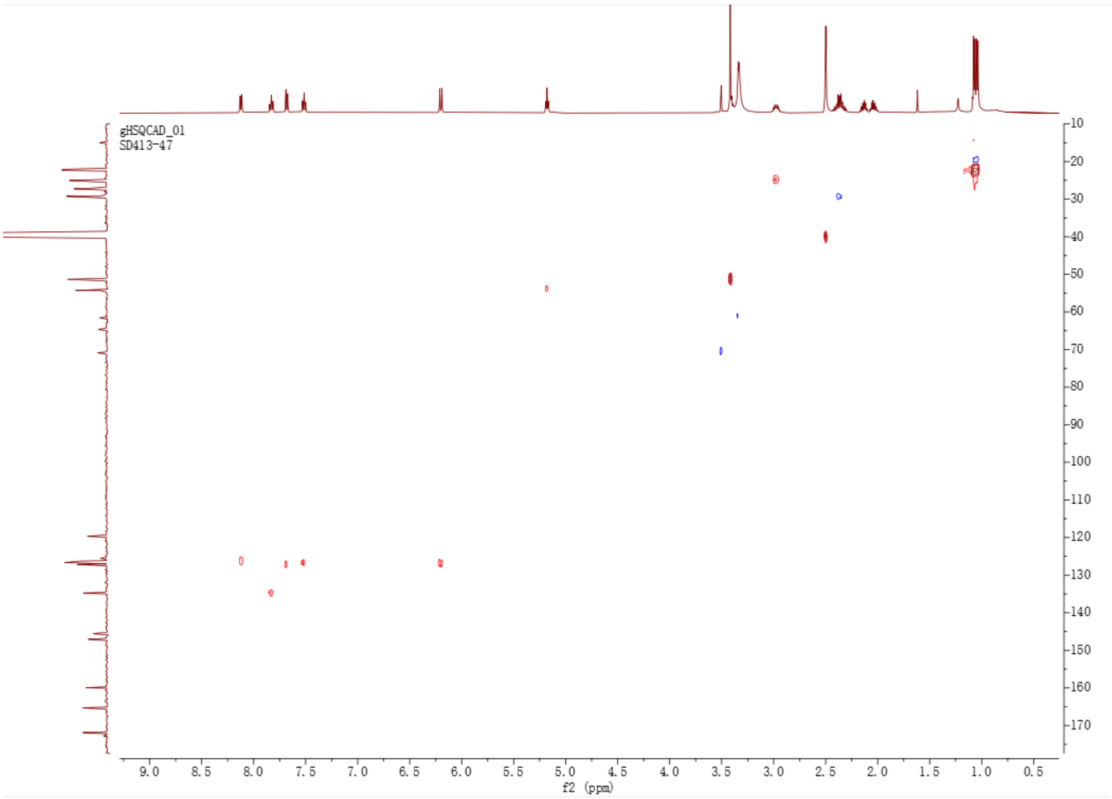

Figure S6. HMBC spectrum of compound **1**.

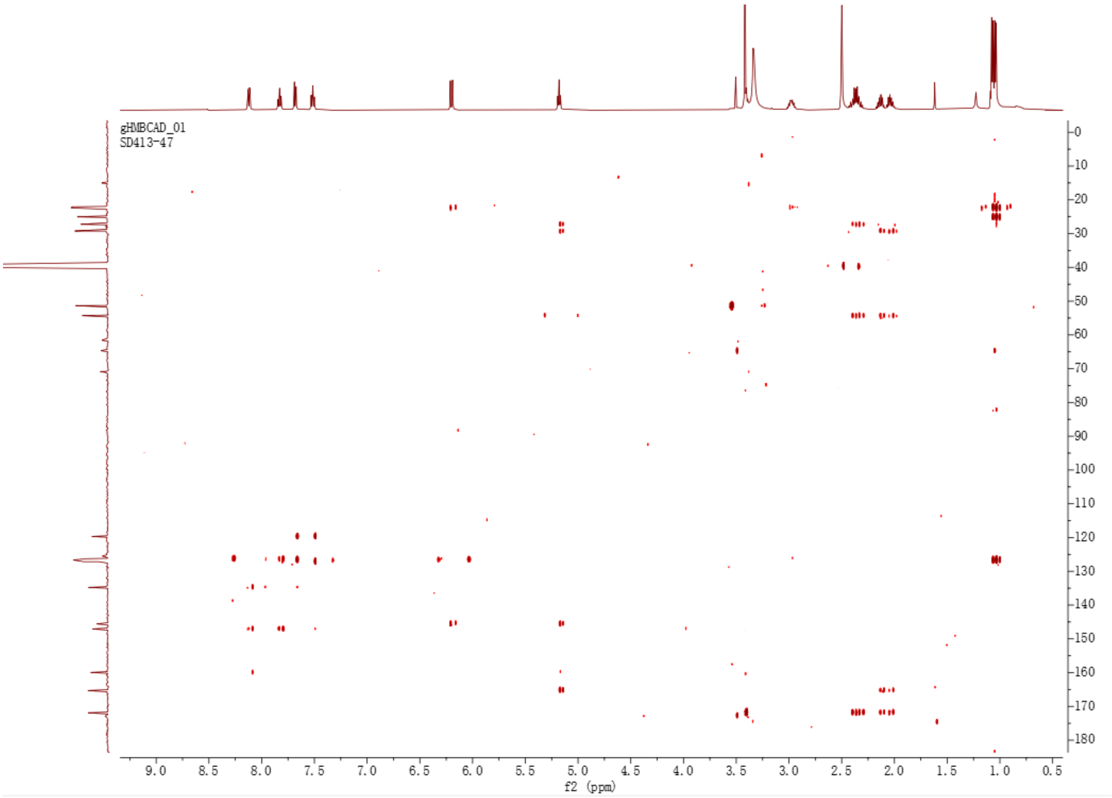

Figure S7. NOESY spectrum of compound **1**.

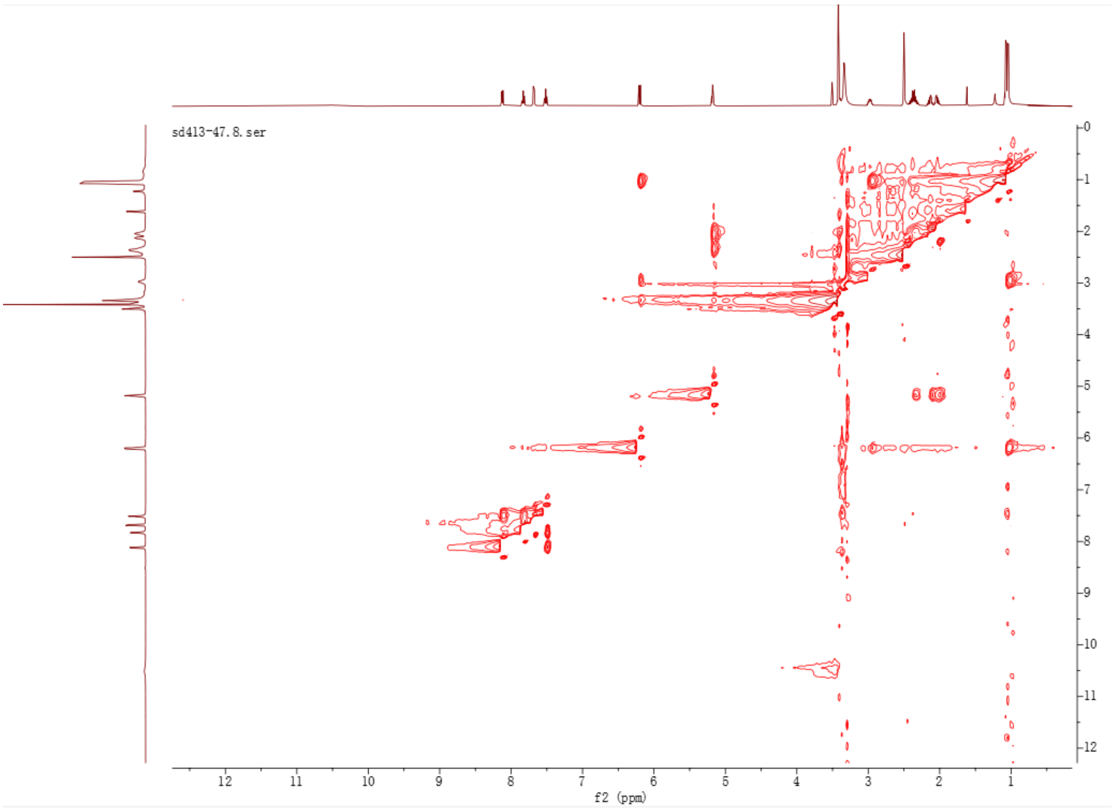

Figure S8. HRESIMS spectrum of compound **2**.

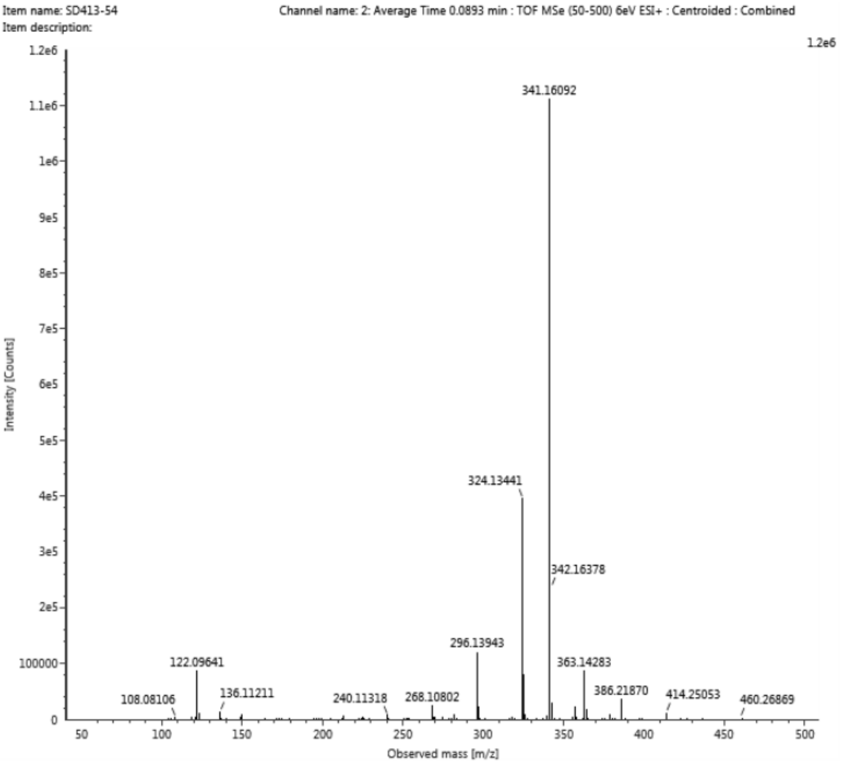

Figure S9. ^{1}H NMR (500 MHz, DMSO-d_6) spectrum of compound **2**.

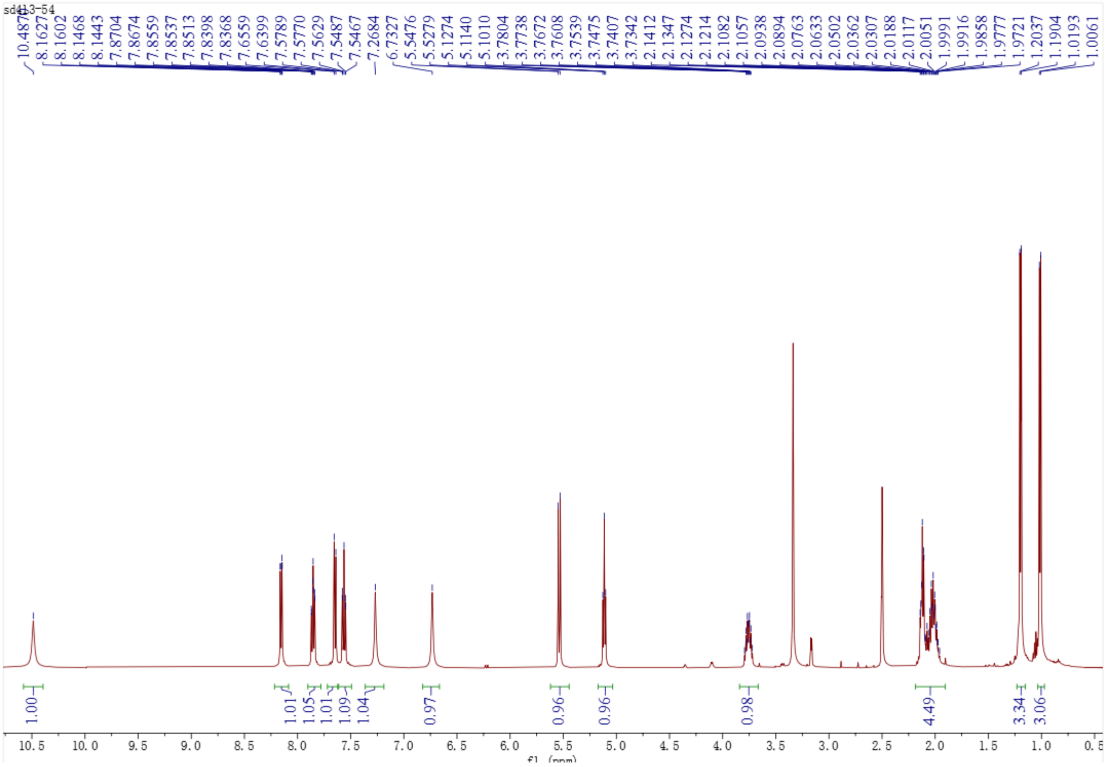

Figure S10. ^{13}C NMR (125 MHz, DMSO-d_6) and DEPT spectra of compound **2**.

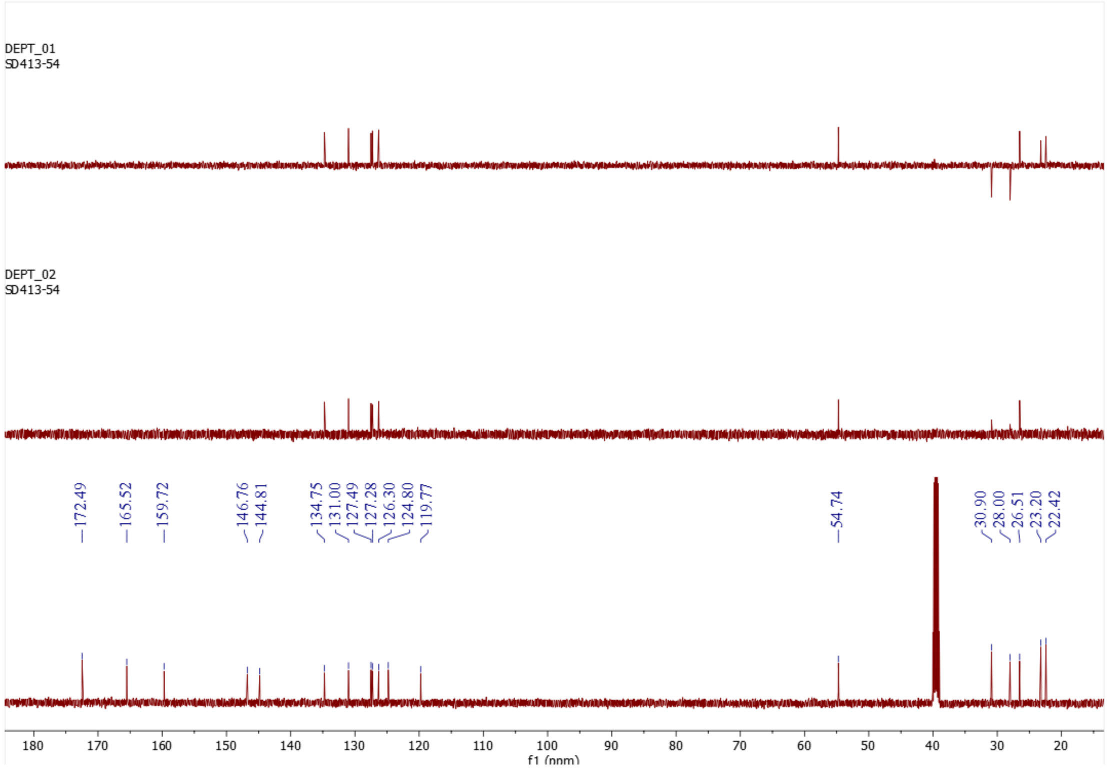

Figure S11. NOESY spectrum of compound **2**.

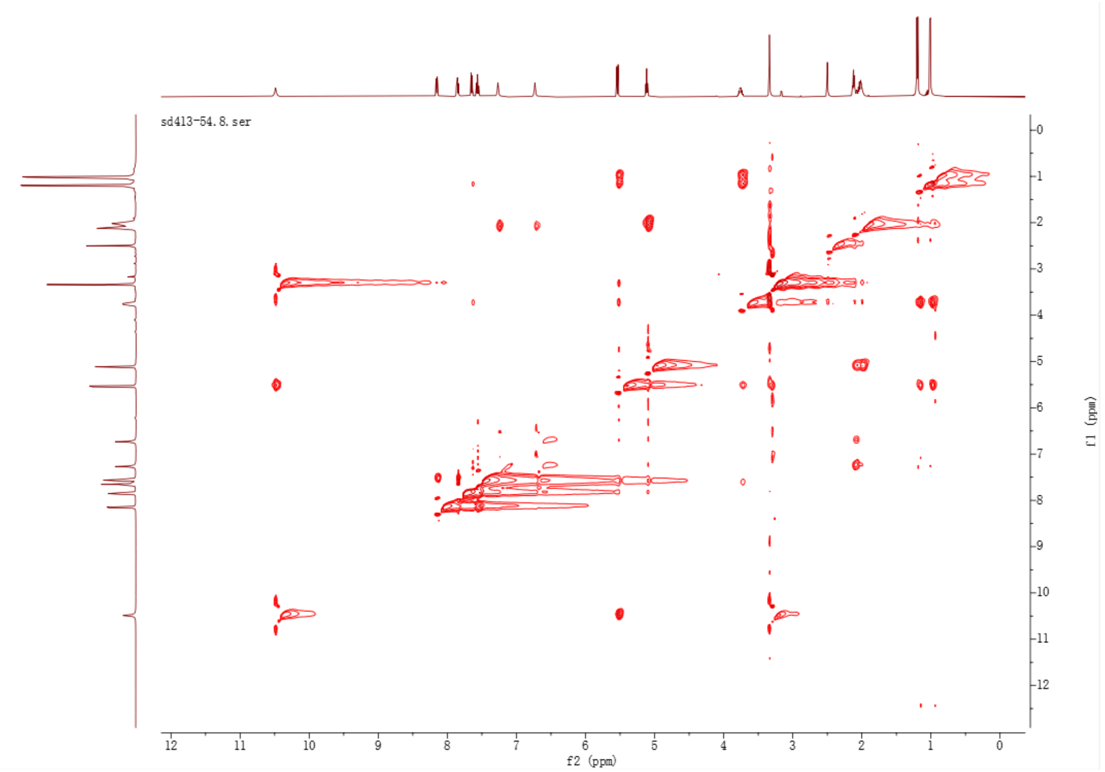

Table S1. ^{1}H and ^{13}C NMR data of compounds **1** and **2**.

No.	**1**		**2**	
	δ_H (*J* in Hz) [a]	δ_C, Type [b]	δ_H (*J* in Hz) [a]	δ_C, Type [b]
1		165.3, C		165.5, C
2-NH	10.51, s		10.49, s	
3		125.5, C		124.8, C
4		145.6, C		144.8, C
6		147.1, C		146.8, C
7	7.68, d (8.1)	127.2, CH	7.65, dd (8.0, 1.3)	127.5, CH
8	7.83, t (7.5)	134.8, CH	7.85, ddd (8.4, 7.2, 1.5)	134.8, CH
9	7.52, t (7.5)	126.7, CH	7.57, ddd (8.4, 7.2, 1.5)	127.3, CH
10	8.12, d (8.1)	126.3, CH	8.15, dd (8.0, 1.3)	126.3, CH
11		119.7, C		119.8, C
12		160.0, C		159.7, C
14	5.18, t (6.5)	54.3, CH	5.11, t (6.6)	54.7, CH
15a	2.04, dt (14.0, 6.9)	27.3, CH_2	2.01, m	28.0, CH_2
15b	2.13, dt (14.0, 6.9)			
16	2.36, m	29.2, CH_2	2.12, m	30.9, CH_2
17		171.9, C		172.5, C
18	6.20, d (10.3)	126.7, CH	5.54, d (9.8)	131.0, CH
19	2.98, m	25.0, CH	3.76, m	26.5, CH
20	1.04, d (6.6)	22.1, CH_3	1.01, d (6.6)	22.4, CH_3
21	1.07, d (6.6)	22.4, CH_3	1.20, d (6.6)	23.2, CH_3
17-OMe	3.42, s	51.3, CH_3		
17-NH_2			6.73, s; 7.27, s	

[a] Measured at 500 MHz in DMSO-d_6; [b] Measured at 125 MHz in DMSO-d_6.

Figure S12. HRESI mass spectrum of compound **4**.

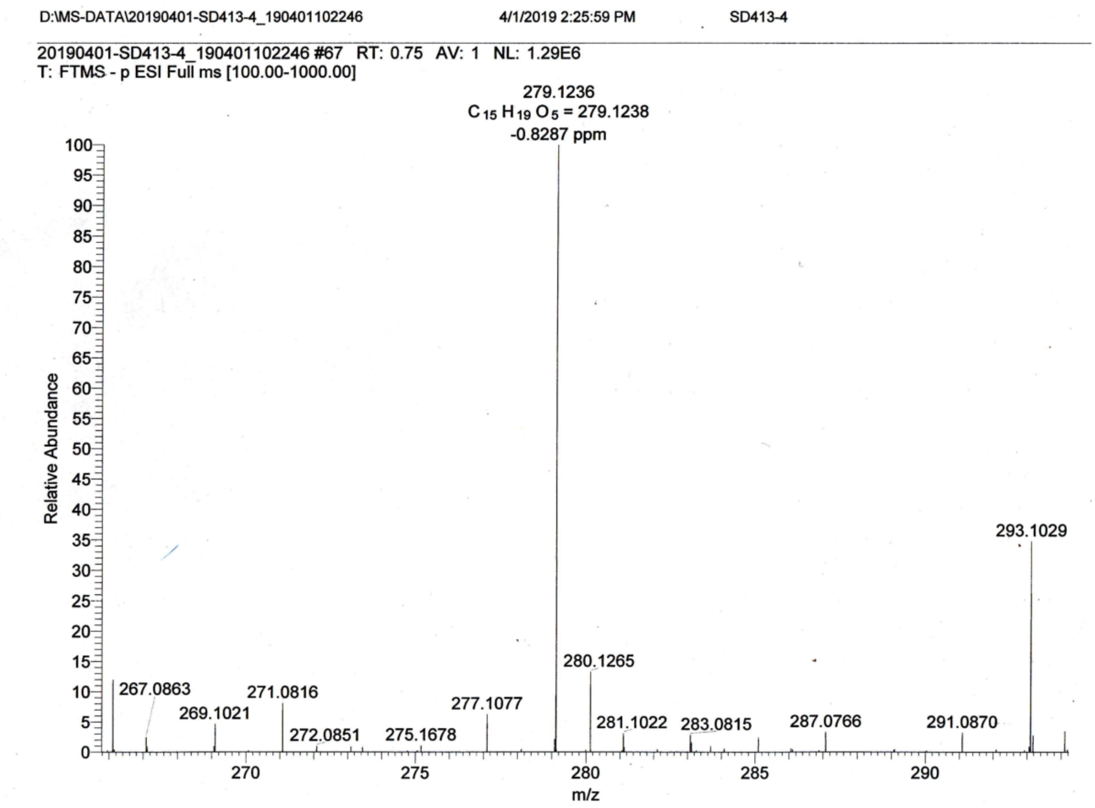

Figure S13. ^{1}H NMR (500 MHz, DMSO-d_6) spectrum of compound **4**.

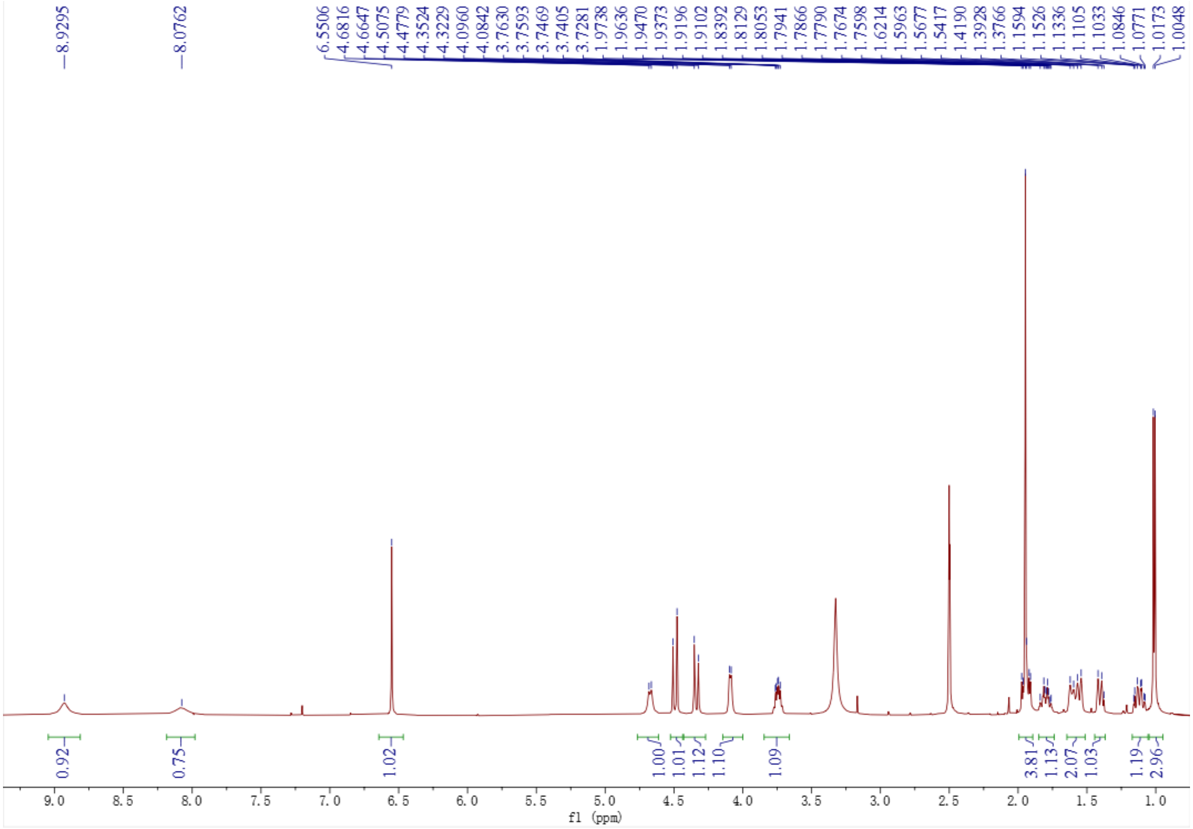

Figure S14. ^{13}C NMR (125 MHz, DMSO-d_6) and DEPT spectra of compound **4**.

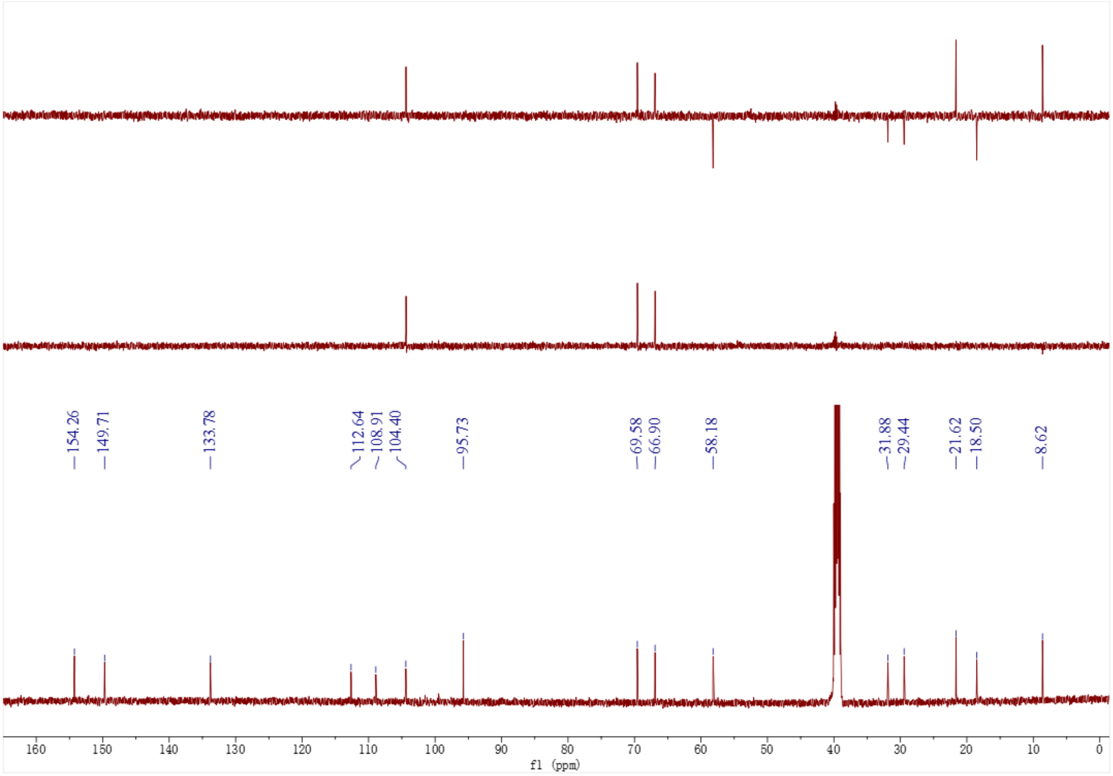

Figure S15. COSY spectrum of compound **4**.

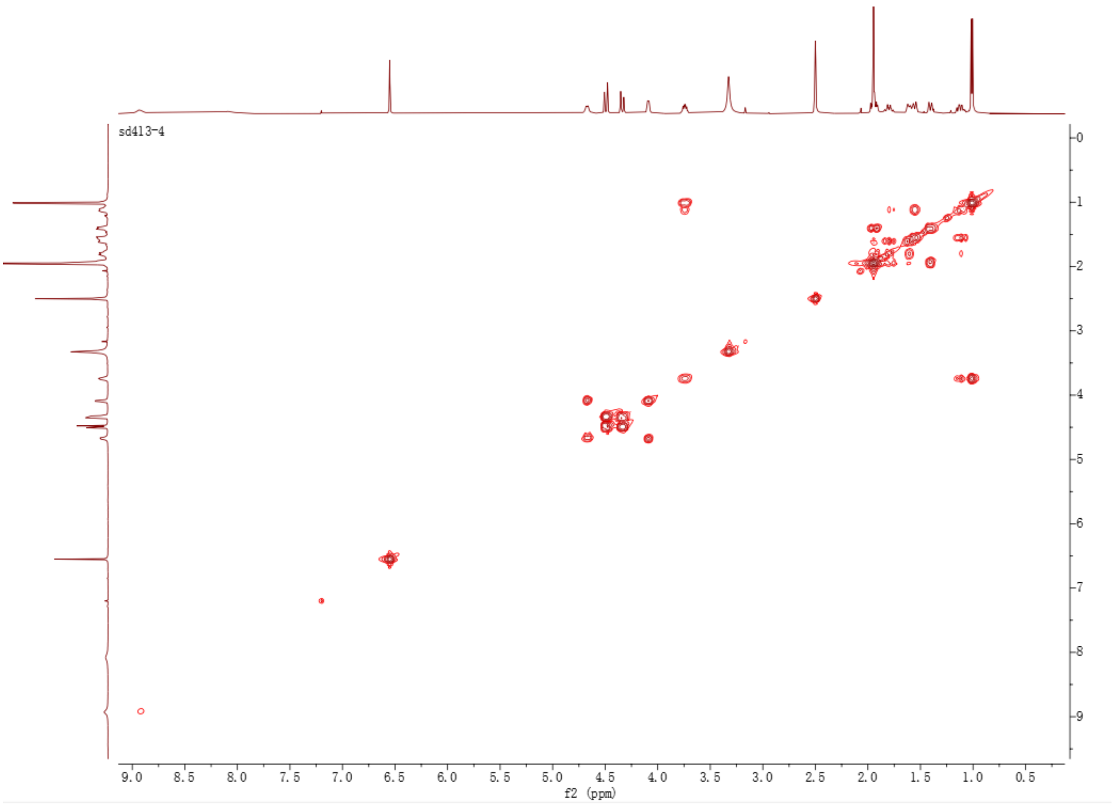

Figure S16. HSQC spectrum of compound **4**.

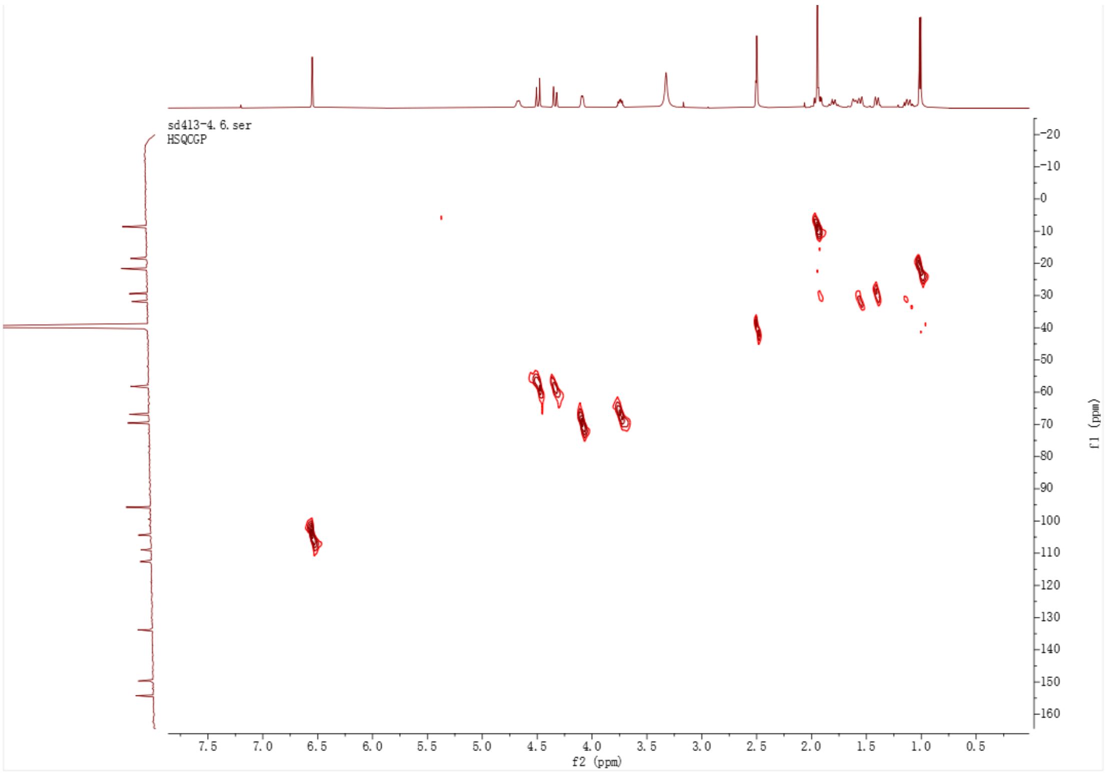

Figure S17. HMBC spectrum of compound **4**.

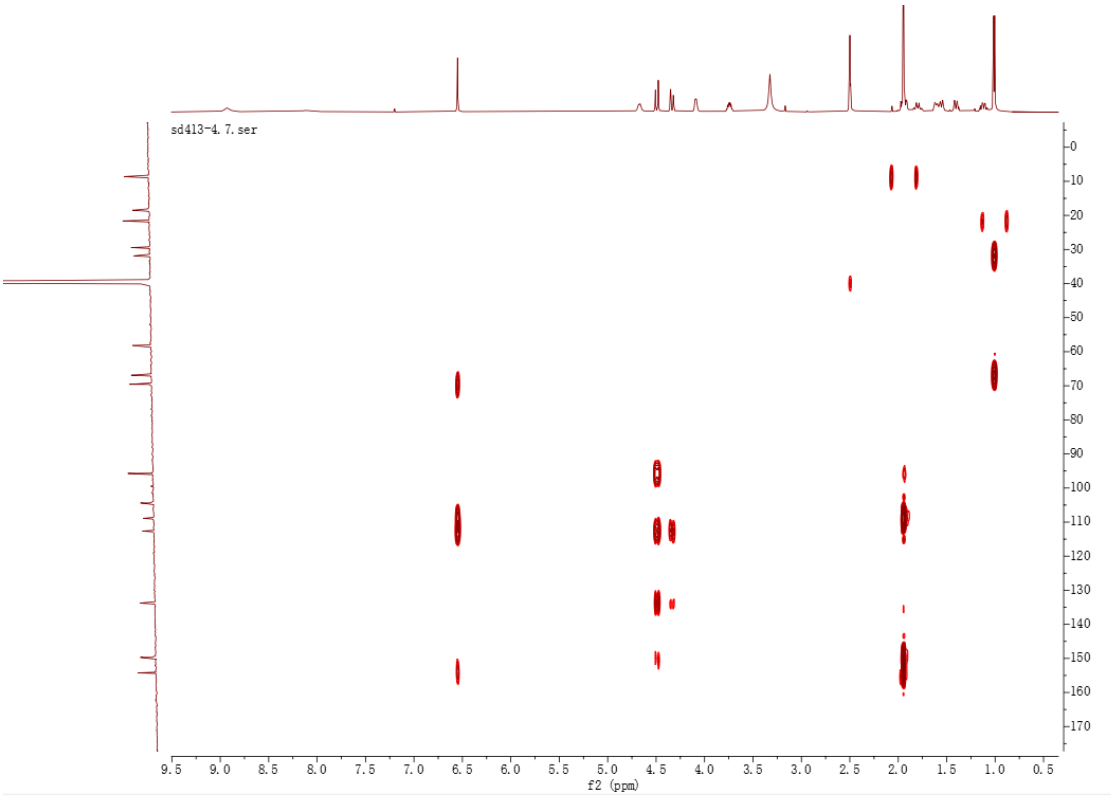

Figure S18. NOESY spectrum of compound **4**.

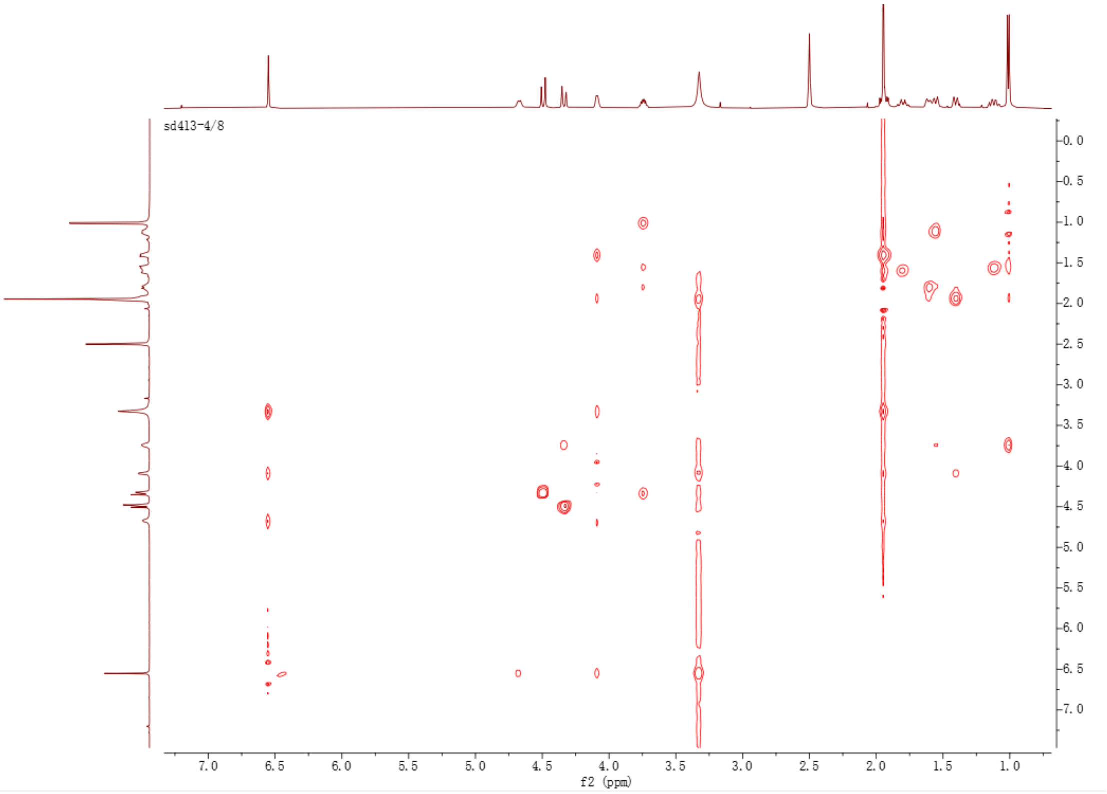

Figure S19. HRESIMS spectrum of compound **5**.

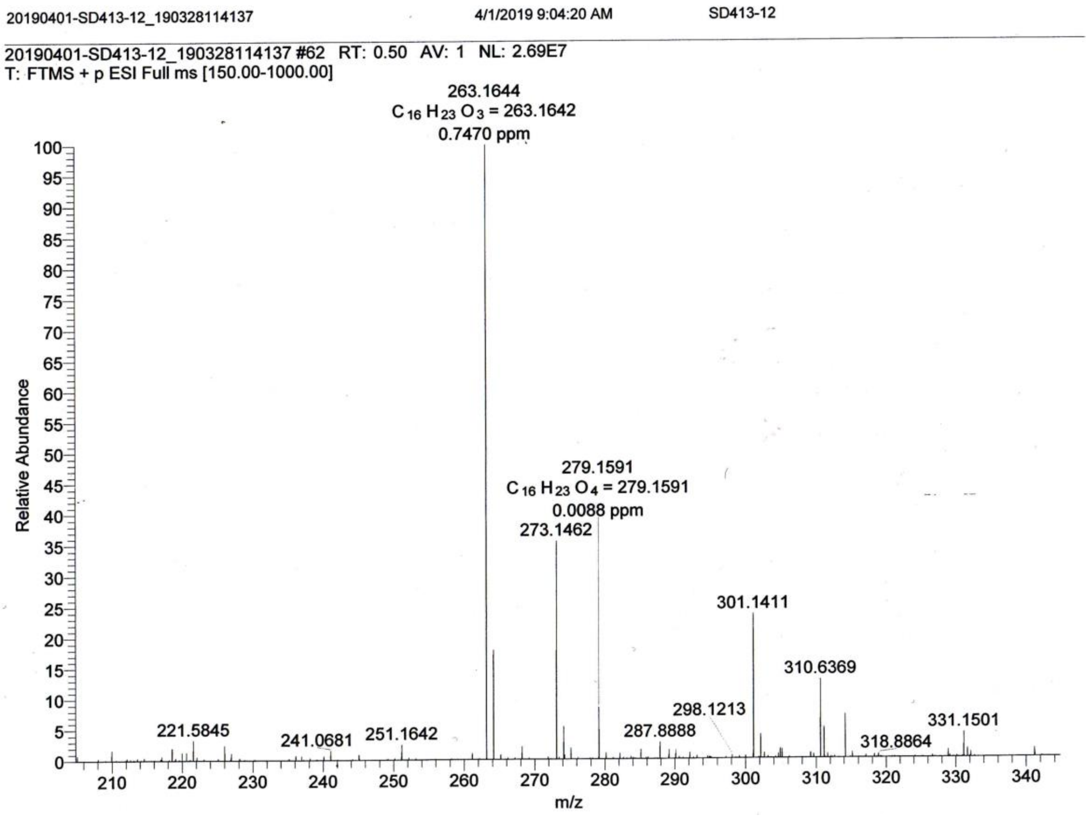

Figure S20. ^{1}H NMR (500 MHz, DMSO-d_6) spectrum of compound **5**.

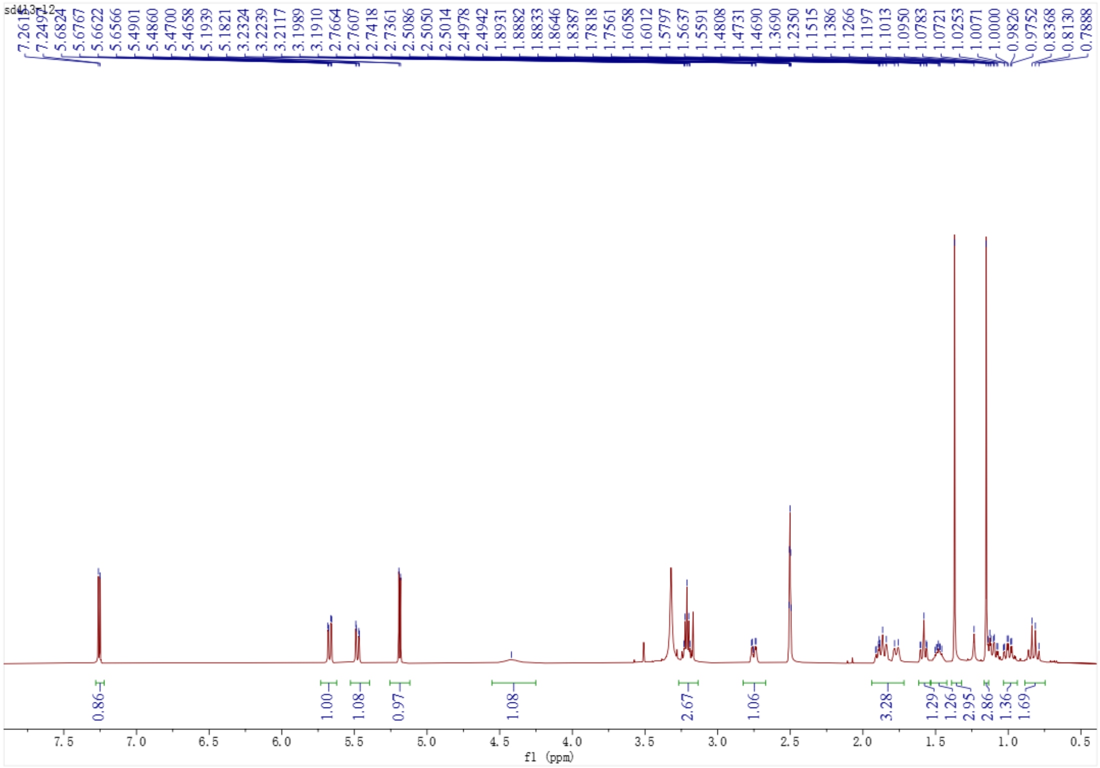

Figure S21. ^{13}C NMR (125 MHz, DMSO-d_6) and DEPT spectra of compound **5**.

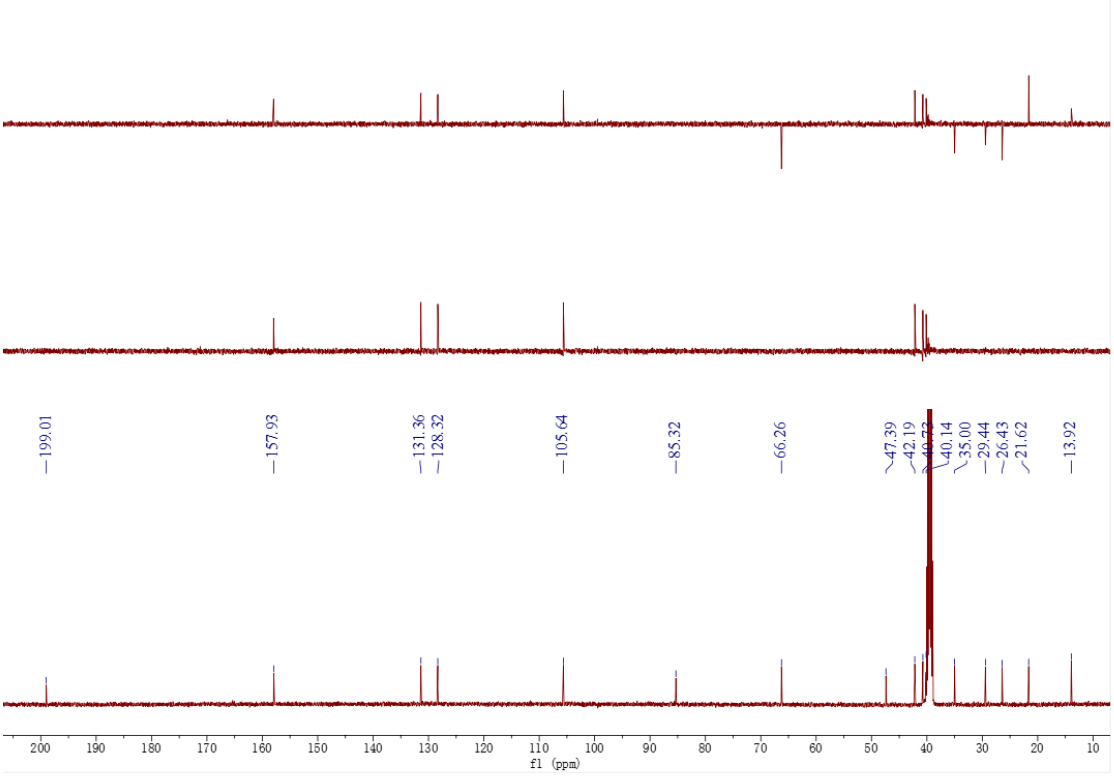

Figure S22. COSY spectrum of compound **5**.

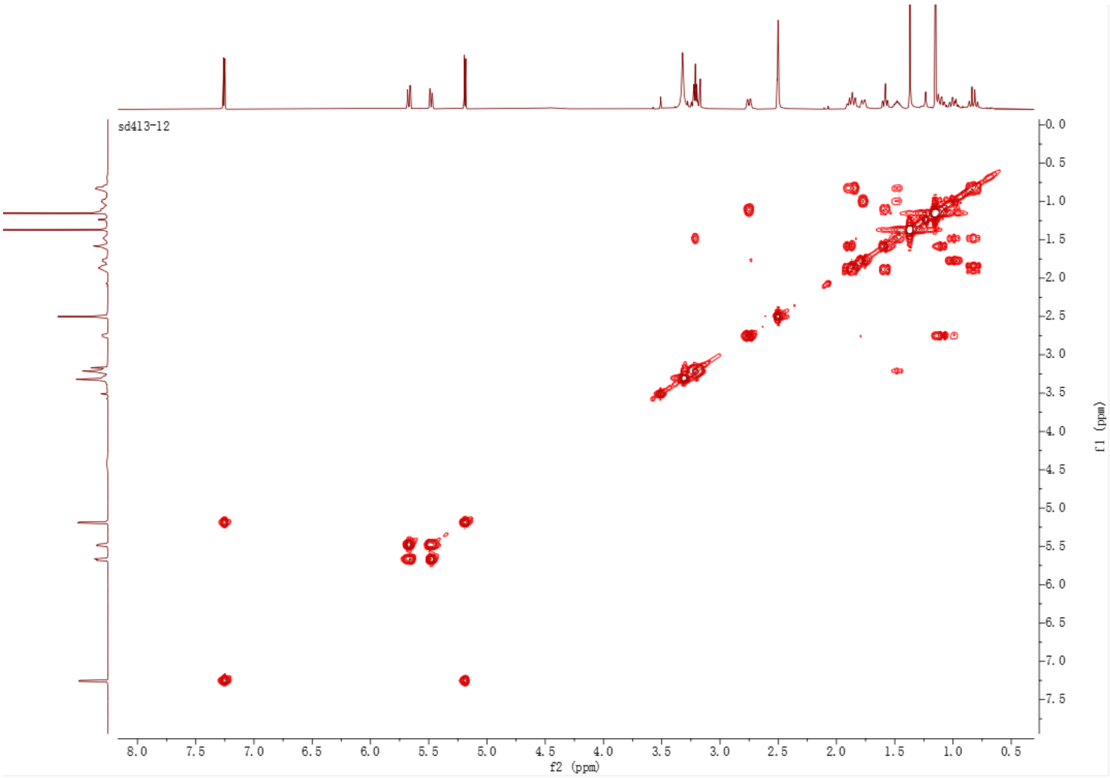

Figure S23. HSQC spectrum of compound **5**.

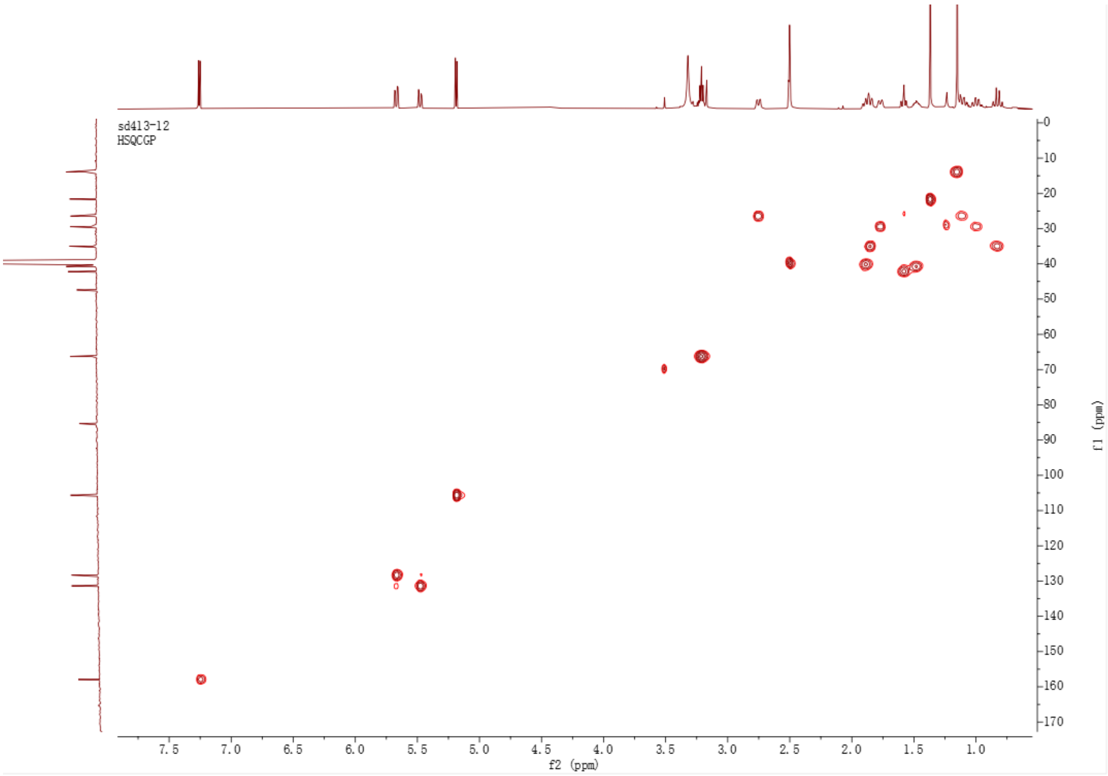

Figure S24. HMBC spectrum of compound **5**.

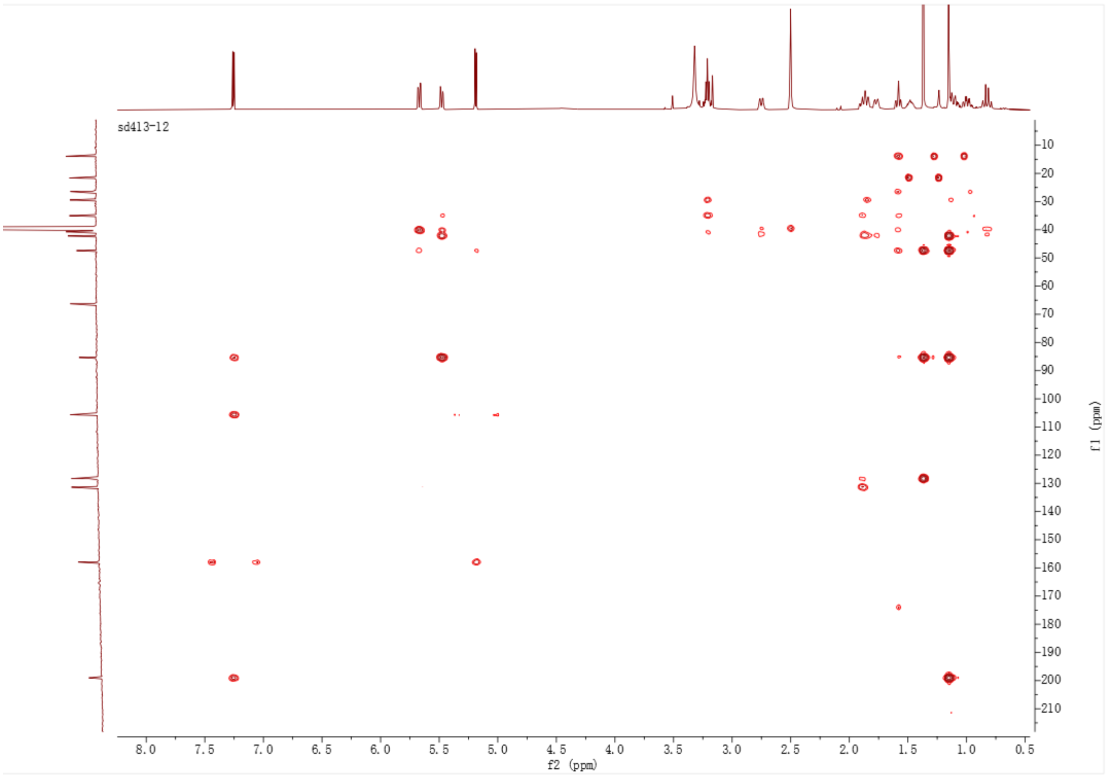

Figure S25. NOESY spectrum of compound **5**.

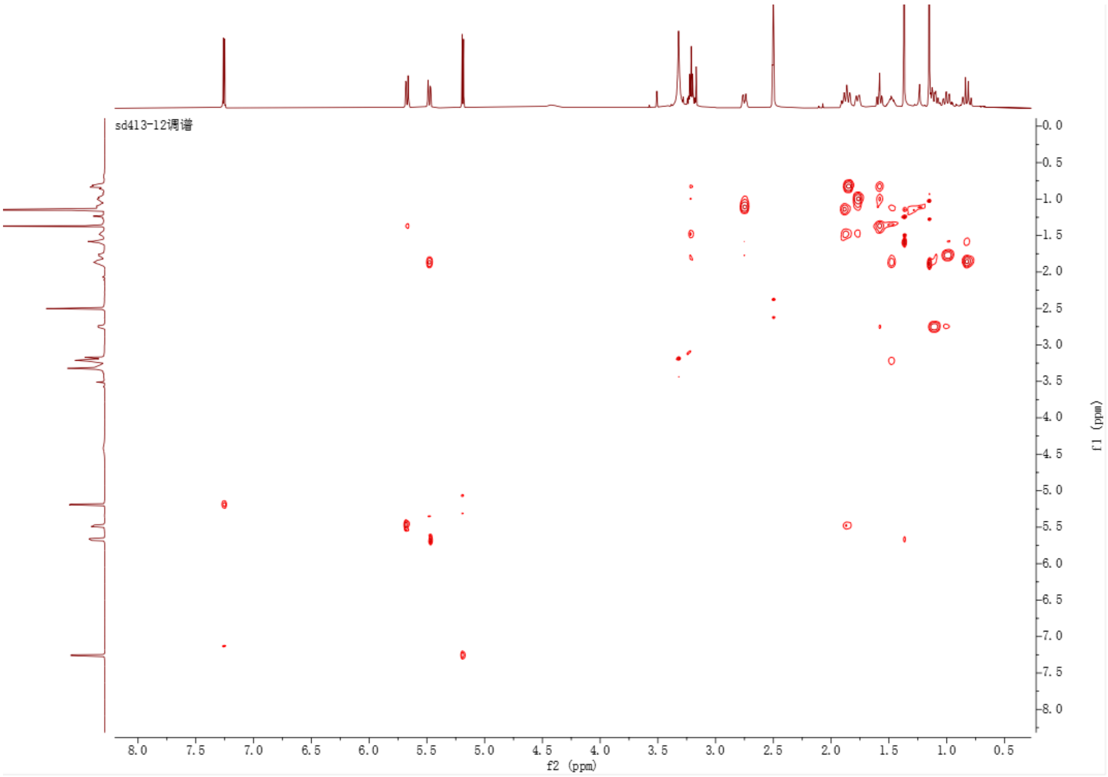

Supplement: Supplementary file 1 [file marinedrugs-18-00553-s001.pdf]
